# Prognostic Role of Neutrophil/Lymphocyte Ratio in Resected Gastric Cancer: A Systematic Review and Meta-analysis

**DOI:** 10.6061/clinics/2018/e360

**Published:** 2018-06-12

**Authors:** Daniel Jose Szor, Andre Roncon Dias, Marina Alessandra Pereira, Marcus Fernando Kodama Pertille Ramos, Bruno Zilberstein, Ivan Cecconello, Ulysses Ribeiro

**Affiliations:** Instituto do Cancer do Estado de Sao Paulo (ICESP), Hospital das Clinicas HCFMUSP, Faculdade de Medicina, Universidade de Sao Paulo, Sao Paulo, SP, BR

**Keywords:** Gastric Cancer, Neutrophil-Lymphocyte Ratio, Meta-Analysis

## Abstract

High levels of inflammatory markers and the neutrophil-lymphocyte ratio appear to be associated with worse overall survival in solid tumors. However, few studies have analyzed the role of the neutrophil-lymphocyte ratio in gastric cancer patients scheduled to undergo curative resection. In the present study, a systematic review and meta-analysis was performed to analyze the relationship between the neutrophil-lymphocyte ratio and overall survival in patients with gastric cancer submitted to curative resection and to identify the clinicopathological features (age, gender, tumor depth, nodal involvement and tumor differentiation) that are correlated with high neutrophil-lymphocyte ratios. A literature search of PubMed, Scopus, Cochrane and EMBASE through November 2017 was conducted. Articles that included gastric cancer patients submitted to curative resection and preoperatory neutrophil-lymphocyte ratio values were included. A total of 7 studies comprising 3264 patients from 5 different countries were included. The meta-analysis revealed an association of high neutrophil-lymphocyte ratios with older age, male gender, lower 5-year overall survival, increased depth of tumor invasion, positive nodal involvement but not with histological differentiation. Evaluation of the neutrophil-lymphocyte ratio is a cost-effective method that is widely available in preoperatory settings. Furthermore, it can effectively predict prognosis, as high values of this biomarker are related to more aggressive tumor characteristics. This ratio can also be used to stratify risk in patients within the same disease stage and may be used to assist in individualized follow-up and treatment.

## INTRODUCTION

Gastric cancer is one of the most frequent solid neoplasias with an aggressive behavior, as only 25% of diagnosed patients can undergo curative resection 1. Overall survival (OS) is low, and efforts to improve diagnosis to avoid late presentation are needed. Clinical staging is performed through endoscopic ultrasound and computed tomography 2. The discovery of novel serum prognostic indicators could complement clinical staging and aid in predicting tumor aggressiveness.

Inflammation is recognized as part of the development and progression of neoplasms, and thus, estimating inflammation through circulating inflammatory cells can indicate systemic inflammatory status and indirectly reflect the severity and prognosis of the neoplasm 3,4. Systemic inflammation can be measured through inflammatory markers, such as the neutrophil-lymphocyte ratio (NLR) 5.

The first study relating NLR and gastric cancer was published in 1998 6. Thereafter, several studies have shown that a high NLR is associated with worse prognosis in gastric cancer patients 7,8. NLR was demonstrated as an independent prognostic factor for worse OS and disease-free survival in 2010 9. It has been estimated that there is a 10% increased risk of death due to gastric cancer for each one-point increase in NLR 10. Nonetheless, few studies have analyzed the impact of NLR in gastric cancer patients submitted to curative resection.

The primary aim of this meta-analysis was to investigate whether high NLR values are correlated with 5-year OS. The secondary aim was to observe the relationship between NLR and various clinico-pathological characteristics, including age, gender, tumor depth (pT), nodal involvement (pN) and tumor differentiation.

## METHODS

### Database search

This study was guided by the Preferred Reporting Items for Systematic Reviews and Meta-Analyses (PRISMA) statement 11. We conducted a literature search of PubMed, Scopus, EMBASE and Cochrane through November 2017. The search algorithm was (gastric cancer OR gastric adenocarcinoma OR stomach neoplasm OR gastric carcinoma) AND (neutrophil-to-lymphocyte ratio OR neutrophil-lymphocyte ratio OR NLR) AND (gastrectomy OR gastric surgery). Articles were also identified using the “related articles” function in PubMed. The bibliographies of relevant articles were individually examined to identify additional studies.

Only articles in English were considered. Case reports, editorials, conference abstracts and reviews were excluded. Articles that included metastatic disease were also excluded. All studies evaluating the prognostic value of NLR in patients with gastric cancer who were submitted to potentially curative gastrectomy were included.

The quality of the studies was assessed using the Newcastle-Ottawa Scale (NOS). In terms of study quality, the cohort studies were considered to be of fair (scores of 4-6) to good (scores of 7-9) quality.

### Data extraction

Two reviewers independently assessed articles for eligibility; discrepancies were resolved by consensus. The following data were retrieved from each study: 1st author, publication year, country, number of patients, study design, cutoff value used to define high NLR, method to obtain the cutoff value, percentage of high NLR patients, presence of neoadjuvant treatment, age, gender, tumor depth, nodal involvement and 5-year OS.

### Statistical analysis

Data were combined into a meta-analysis using RevMan 5.3 analysis software (Cochrane Collaboration, Copenhagen, Denmark). The hazard ratio (HR) and 95% confidence interval (CI) were represented graphically using a Forest plot. The publication bias was assessed by Funnel plots. The I^2^ was graded as low (I^2^<25%), moderate (I^2^=25 to 75%) or high (I^2^>75%) to determine the heterogeneity between studies. All statistical tests were two-sided and a *p*-value <0.05 was considered statistically significant.

## RESULTS

### Study characteristics

**Figure 1:**
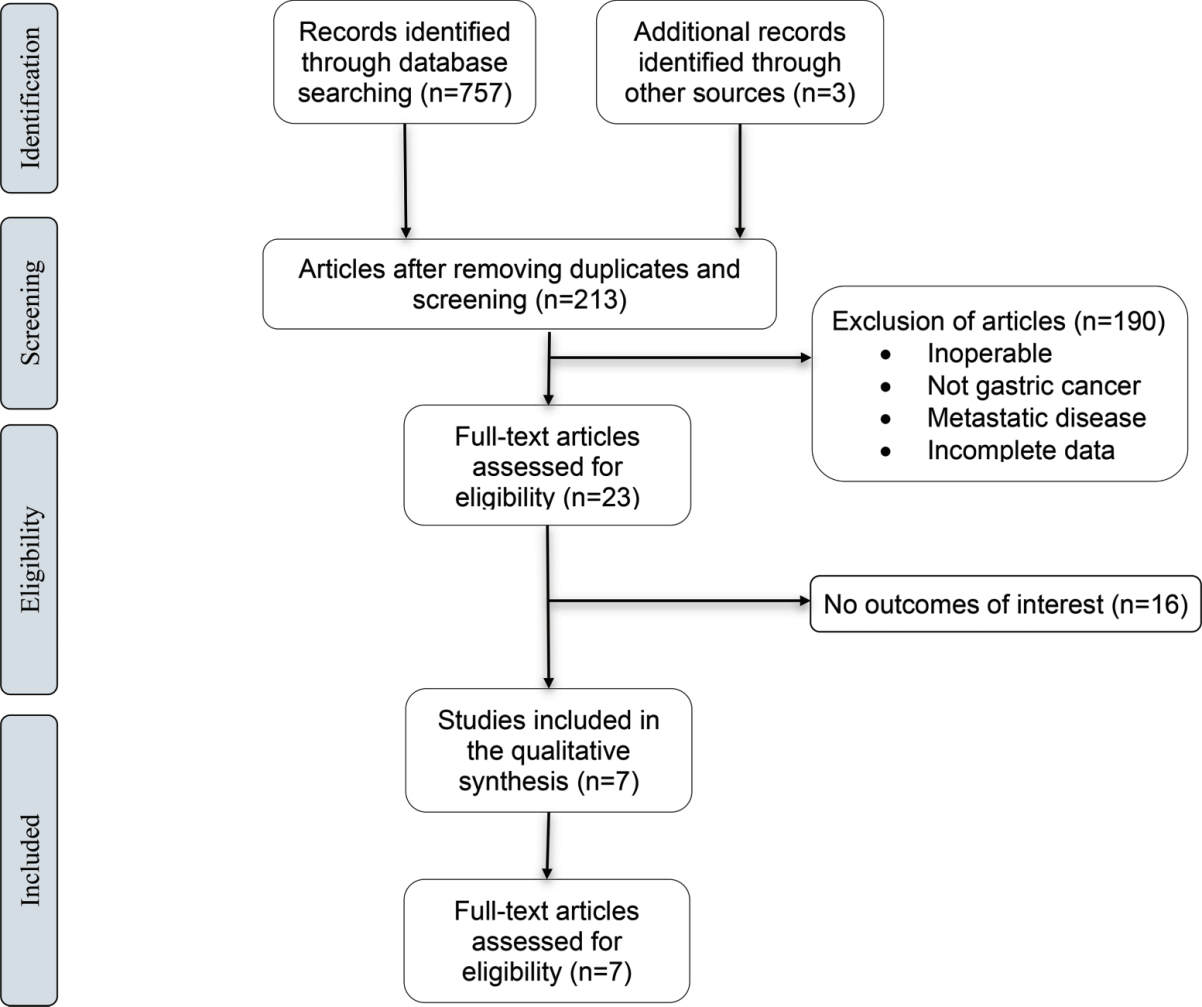
Literature screening flow chart and results.

The search strategy generated 757 references from PubMed (n=110), EMBASE (n=72), Cochrane Library (n=3) and Scopus (n=572). We excluded articles if they lacked information about OS, did not analyze the prognostic value of NLR, or were replicated across the different search platforms. In total, 7 eligible trials were identified and reviewed (Figure 1). Cumulatively, 3,264 gastric cancer patients submitted to potentially curative gastrectomy were evaluated.

The median quality score of the involved studies was 6.8 (range 6 to 8). Of the 7 studies, 6 were defined as good, and 1, as fair according to the NOS (Table 1).

All 7 studies were cohort studies, comprising 1 prospective study and 6 retrospective studies, which were published between 2010 and 2015 and were performed in 5 different countries.

The mean NLR cutoff value was 2.97 with a range of 1.4 to 4.02. Different methods were used to define NLR cutoff values, including the receiver operating characteristic (ROC) curve, the median and the 75^th^ percentile. High NLR values were defined as NLR values equal to or above the cutoff. A summary of the study characteristics is shown in Table 2.

### Neutrophil-lymphocyte ratio and clinicopathological factors

**Figure 2:**
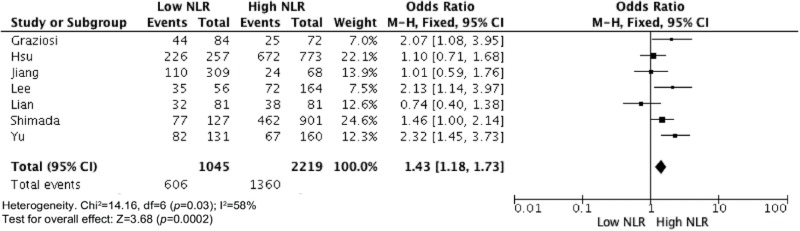
NLR and age.

**Figure 3:**
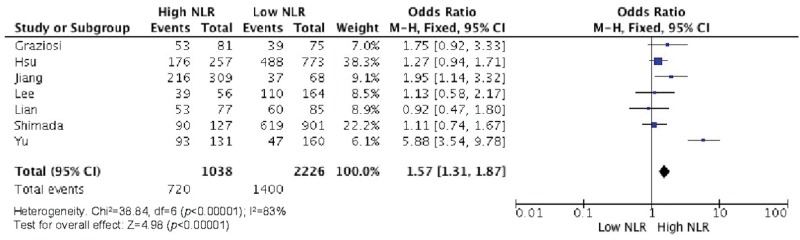
NLR and gender.

**Figure 4:**
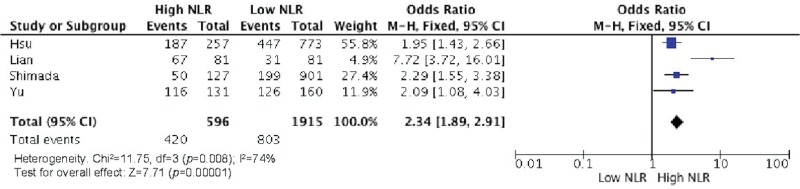
NLR and pT status (pT3 and pT4).

**Figure 5:**
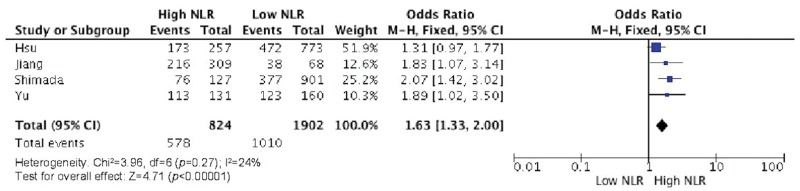
NLR and lymph node involvement.

**Figure 6:**
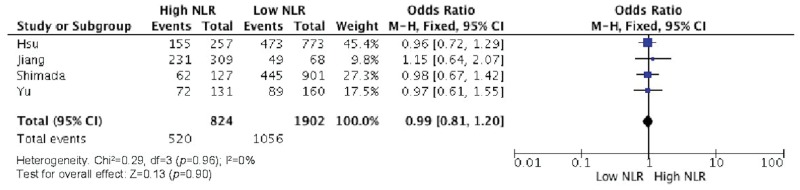
NLR and tumor differentiation grades.

Elevated NLR values were associated with older age (Figure 2), male gender (Figure 3), deeper wall invasion (pT3 and pT4) (Figure 4), positive lymph node involvement (Figure 5), but not with tumor differentiation (Figure 6).

Significant relationships of higher NLR values with pN and grade with non-significant heterogeneity (I^2^=24%, *p*=0.27) were detected according to our pooled estimates (Figure 5).

There was evidence of moderate heterogeneity among studies concerning the association of NLR with age and pT (I^2^=58%, *p*=0.03 and I^2^=74%, *p*=0.008, respectively) (Figures 2 and 4) and evidence of high heterogeneity for studies involving gender and NLR (I^2^=83%, *p*<0.0001) (Figure 3).

Regarding nodal status, although higher NLR values were associated with lymph node positivity, the number of positive lymph nodes was not associated with higher NLR values. Interestingly, Hsu et al. 12 compared NLR with lymph node ratio (metastatic lymph nodes/total lymph nodes) and observed that higher NLRs were correlated with higher lymph node ratios.

### Neutrophil-lymphocyte ratio and overall survival

**Figure 7:**
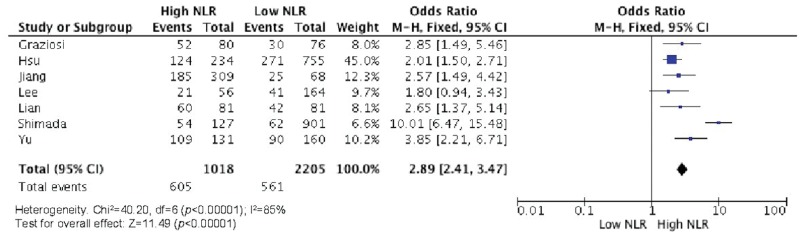
NLR and overall survival.

One study compared OS in 1, 3 and 5 years among high and low NLR groups, reporting OS of 85.4%, 67.1% and 62.5% *vs* 93.2%, 82.4% and 74.4%, respectively 9. Hsu et al. 12 showed lower 3- and 5- year OS rates in patients with high NLRs than in those with low NLRs (55.1% *vs* 71% and 47.2% *vs* 64.1%). All studies showed lower 5-year OS in patients with high NLRs (Figure 7).

Two studies stratified risk of death in patients of the same disease stage 13,14. Both showed that stage II and III patients presented different prognosis according to NLR, for which high NLR predicted worse prognosis. These results differed from those of stage I patients, as Yu et al. 14 did not observe any difference in prognosis according to NLR in stage I patients.

## DISCUSSION

Several articles have been published in the literature regarding the prognostic impact of NLR in patients with solid tumors 15. This meta-analysis focused on the impact of NLR in gastric cancer patients submitted to potentially curative resection. A high global NLR has a significant negative impact on survival rate and disease-free survival, reflecting the close relationship between pro-inflammatory systemic status and cancer progression 5.

Indeed, the neoplastic process is mediated by different inflammatory cells, and the combined effect of neutrophilia and lymphopenia results in tumoral development and progression 16. Neutrophils present a pro-tumoral behavior, as they promote angiogenesis, damage DNA, inhibit T-cell activity against tumoral cells, and facilitate the metastatic process 17. Inversely, lymphocytes exert an anti-tumoral function when they recognize tumoral cell antigens, promoting cytolytic activity against these cells 4,18,19.

According to the logistical regression, NLR was an independent risk factor of worse overall and disease-free survival in all but one study included 20. This finding demonstrates that NLR is not simply an indicator of tumor volume and TNM status or a sign of other factors of worse prognosis, such as age. Thus, NLR reflects the inflammatory status of the host and may determine a pro- or anti-tumoral microenvironment. In this context, attempts have been made to correlate NLR with the intra- and peritumoral proportions of inflammatory cells, and peripheral proportions may reflect the tumoral microenvironment 21.

Two aspects require further in-depth study: the method employed to determine the NLR cutoff and the period during which the blood sample is obtained. There is no consensus regarding these aspects, and different techniques are used across studies. The ROC curve and median value are the primary methods used to determine the NLR cutoff value. The ROC curve has the potential to determine a more accurate value, as it can estimate the value that maximizes sensitivity and specificity.

All the studies reported in the present meta-analysis obtained the blood samples preoperatively. In addition, there was no consensus on the timing of acquiring the blood samples, although samples were generally taken within 2 weeks of surgery. Only one study reported the exact hour when the blood test was performed, which confers the advantage of more homogeneous results without interference of circadian variations in blood cells 22. Standardization of the exact hour for blood sample collection is particularly difficult in studies with large populations.

Whether the value of NLR can modify treatment and follow-up remains an unanswered question. Some have advocated that the high-NLR population requires more aggressive treatment regarding chemotherapy and immunotherapy 23. Additionally, as NLR reflects inflammation, the use of anti-inflammatory drugs and anti-tumoral vaccinations might play a role in therapy 24,25.

Recent studies have reported the utilization of prognostic scores, including NLR. There are several prognostic scores based on inflammatory markers or proteins, such as c-reactive protein and albumin, but a score that included NLR was reported to confer better prognostic strength 26,27.

There are some limitations of this meta-analysis. As there is no consensus regarding NLR cutoff or the method employed to calculate NLR, the values vary among studies. Additionally, there was significant heterogeneity among studies. However, because this was a small meta-analysis (including only seven studies), it was not possible to perform meta-regression or subgroup analysis in cases where heterogeneity was significant. Other limitations included the retrospective nature of several articles and the lack of descriptions of underlying diseases or concomitant infection that could somehow affect NLR status. We assume that since patients were submitted to elective surgery, they were in relative good status performance and comorbidities were controlled (patients submitted to emergency surgery were excluded).

In conclusion, evaluation of NLR is an easy and cost-effective method for predicting overall and disease-free survival in gastric cancer patients submitted to curative resection. Furthermore, this method offers the opportunity to individualize treatments by identifying patients with worse prognosis and ensuring they undergo a closer follow-up or receive modified adjuvant therapy.

## AUTHOR CONTRIBUTIONS

Szor DJ was responsible for the project elaboration, manuscript writing and statistical analysis. Dias AR, Pereira MA, Ramos MF were responsible for the manuscript revision. Zilberstein B was responsible for the manuscript elaboration. Cecconello I was responsible for the manuscript revision. Ribeiro-Júnior U was responsible for the manuscript elaboration and revision.

## Figures and Tables

**Table 1:** Quality assessment of included articles based on the Newcastle-Ottawa Scale.

Name	A	B	C	D	E	F	G	H	Score
**Lee et al. (20)**	*	*	*	*	*	*		*	7
**Lian et al. (21)**	*	*	*	*	*	*	*		7
**Graziosi et al. (13)**	*	*	*	*		*	*	*	7
**Yu et al. (14)**	*	*		*	*	*		*	6
**Shimada et al. (9)**		*	*	*	*	*	*		6
**Hsu et al. (12)**	*	*	*	*	*	*	*		7
**Jiang et al. (27)**	*	*	*	*	*	*	*	*	8

A. Representativeness of the exposed cohort; B. Selection of the non-exposed cohort; C. Determination of exposure; D. Demonstration that outcome of interest was not present at the start of the study; E. Comparability of cohorts on the basis of the design analysis; F. Assessment of outcome; G. Sufficient follow-up duration to measure outcomes; H. Adequacy of follow-up cohorts.

**Table 2:** Characteristics of the included studies.

	n	Age (yrs)	Study design	NLR cutoff	Type	% high	Country	Median follow-up (months)	Blood sample	Neoadjuvant	OS 5 yrs low vs high (%)
**Lee et al. 2013 (20)**	220	57 (23-89)	retrospective	2.15	75^th^ percentile	25.4	Korea	NA	Before surgery	NA	74.4 *vs* 62.5
**Lian et al. 2015 (21)**	162	56 (31-82)	retrospective	4.02	Median	47.5	China	NA	Before surgery	NA	48.5 *vs* 26.3
**Graziosi et al. 2014 (13)**	156	74 (39-91)	retrospective	2.34	Median	51.2	Italy	23	Day before surgery	Yes (18)	67 *vs* 46.6
**Yu et al.2015 (14)**	291	NA	retrospective	3.5	NA	45	China	NA	Week Before surgery	No	43.6 *vs* 17
**Shimada et al. 2010 (9)**	1028	65 (26-89)	prospective	4	NA	12.4	Japan	23	Before surgery	NA	81 *vs* 56
**Hsu et al.2015 (12)**	1030	NA	retrospective	3.44	NA	52.8	Taiwan	30	Before surgery	No	64.1 *vs* 47.2
**Jiang et al. 2014 (27)**	377	64 (25-80)	retrospective	1.4	ROC analysis	81.9	China	42	Day before surgery	No	63.2 *vs* 36.9

NA: not available.
